# Extraction, purification, and identification of total saponins from hazel mushroom and its application evaluation

**DOI:** 10.3389/fnut.2025.1737642

**Published:** 2026-01-08

**Authors:** Yuan Fang, Wenjun Kan, Yilizhati Yimamu, Xinyu Cui, Yanan Luo, Xueling Cao

**Affiliations:** 1 School of Laboratory Medicine (Pharmaceutical Sciences), Jilin Medical University, Jilin, China; 2 College of Science, Qiongtai Normal University, Haikou, China

**Keywords:** activity, application, extraction, total saponins of hazel mushroom, UPLC/Q-TOF-MS

## Abstract

A novel approach was formulated for extracting saponins from hazel mushroom (SHM) via microwave-assisted extraction (MAE). Response surface methodology was employed to refine and optimize how extraction parameters affect extraction efficiency. This study examined parameters such as solid–liquid ratio (1:15–1:30 g/mL), extraction power (from 200 to 600 W), extraction duration (between 5 and 25 min), and ethanol concentration (from 40 to 80%). The identified optimal extraction parameters were as follows: ethanol concentration of 57.52%, solid-to-liquid ratio of 1:25 g/mL, extraction time of 20.00 min, and extraction power of 369.75 W. When these ideal conditions were applied, the extraction yield of SHM reached a notable 34.61%. Using UPLC/Q-TOF-MS, 22 compounds were identified from the SHM obtained from MAE, primarily consisting of sesquiterpenes, adenosine, sterols, purines, and diterpenes. Finally, the anticancer effects of SHM were analyzed. The results indicated that SHM exhibited anti-tumor activity and antioxidant activity. For the first time, this research conducted an in-depth analysis of the total saponin extract derived from hazel mushroom. The findings of this study may offer a valuable methodology for optimizing the extraction process, ensuring quality control, and evaluating bioactivities. This is the first systematic study on saponins from hazel mushroom.

## 1 Introduction

For centuries, natural medicinal herbs have been extensively applied in the treatment of many diseases in China and other Asian countries (1–4). Hazel mushroom is classified under the genus *Tricholoma*, which is a medicinal food-related fungus prevalent in North America, Europe, and Asia (5, 6). In recent years, the chemical composition and biological activity of hazel mushroom have attracted increasing attention. To date, about 100 compounds, such as terpenoids, sterols, adenosine, organic acids and polysaccharides, have been successfully isolated and accurately characterized from hazel mushroom (5, 7, 8). However, the important saponins in hazel mushroom (SHM) have not yet to be reported. The fungus is valued for its content of fiber, proteins and functional compounds like schizophyllan, saponins and ergosterol (9). Saponins are mainly composed of hemiacetal hydroxyl groups of sugars and aglycones of non-sugar parts (10, 11). Saponins can be divided into two categories based on their glycogen carbon skeletons, namely, steroidal saponins and triterpene saponins (12). As a non-toxic bioactive substance, saponins have many biological effects, including anti-tumor, blood lipid lowering, anti-oxidation, antibacterial, and anti-inflammatory properties (13–16). They can be used in medical treatment, medicine, and functional food (17, 18).

Saponins are complex glycosides found in higher plants and some marine organisms. Previous studies have reported that triterpenoid saponins predominantly occur in several valuable traditional Chinese medicinal substances, such as *Panax ginseng*, *Panax notoginseng*, *Platycodon grandiflorum*, Radix *Codonopsis* and Radix *Polygonae*. Steroidal saponins are mainly found in plants such as the Liliaceae, Dioscorea, and Solanaceae families (10). Saponins, recognized as a green and non-toxic bioactive compound, are reported in the literature to exhibit numerous biological functions, including antitumor, lipid-lowering, antioxidant, antibacterial, and anti-inflammatory properties. They can also be used in medical treatment, pharmaceuticals, and functional foods. Therefore, we hypothesized that SHM possess similar biological activities, aiming to optimize the utilization of hazel mushroom resources. This study investigated the presence of saponins in hazel mushroom, as well as their extraction and activity.

Given the biological activity of saponins, there is an urgent need to explore and examine the extraction and purification of SHM. To extract saponins from alternative medicinal plants, researchers often employ three methods of extracting saponins, namely, Heat Reflux Extraction (HRE), microwave-assisted extraction (MAE), and Ultrasound-assisted extraction (UAE) (19–21). HRE, as a traditional strategy, offers straightforward performance, easy installation, and stabilization for industrial production. Its most attractive feature is that it can extract saponins of varying polarity by changing the polarity of the extraction solvent. MAE, as an advanced technique, offers numerous benefits, which include elevated extraction efficiency, minimal solvent usage, high-purity extracts, and reduced extraction duration. Compared to the other two methods, the MAE method saves time and consumes less energy in terms of extraction time. Generally, the MAE method takes 10 min to achieve maximum yield, while the HRE method takes 90 min. The UAE method takes approximately 30 min. Collectively, these properties make it an optimal choice for extracting bioactive chemicals from plant-based materials. Thus, MAE was employed and fully investigated to extract SHM in the present work. This paper is the first report of saponins being extracted from hazel mushroom via MAE.

Response Surface Methodology (RSM) is a statistical experimental design technique specifically designed for optimizing biological processes. Its prevalent use involves identifying optimal parameters via the establishment of a continuous variable surface model, facilitating the evaluation of factors that influence extraction processes (22, 23). Moreover, the number of experimental groups has been significantly decreased, conserving time and resources. Currently, this approach has been successively utilized to optimize several extraction processes. This work employed RSM to optimize MAE conditions.

Furthermore, the composition of saponin-rich hazel mushroom extract should be identified to elucidate the chemical basis related to its pharmacological properties. UHPLC-Q-TOF-MS has proven to be an effective method for the rapid qualitative analysis of bioactive components in herbal medicines (24). UHPLC provides rapid, high-resolution separation and improved sensitivity through ultra-high pressure. Q-TOF MSE, a high-resolution tandem mass spectrometry technique, provides mass spectra with exceptional sensitivity, accuracy, and precision. It also facilitates component identification by analyzing fragmented ions resulting from collision-induced dissociation. Thus, the biological activities of SHM extracted by MAE were estimated, and the relevant results were elucidated based on the chemical components.

Accordingly, this research aimed to develop a simple yet efficient extraction approach for SHM via MAE, with RSM employed for method optimization. UHPLC-Q-TOF-MS was applied to characterize these saponins in the extract. Moreover, the *in vitro* antitumor activity of SHM was examined. We hypothesized that MAE-extracted SHM saponins exhibit strong cytotoxic and antioxidant activities.

## 2 Materials and methods

### 2.1 Instruments and reagents

High-performance liquid chromatography (HPLC, 1290 series) and ion trap mass spectrometry were conducted using instruments from Agilent (Palo Alto, CA, United States). Ultraviolet spectrophotometry (TU − 1950, Beijing General Analysis Instrument Co. LTD.) and flow cytometry (A00 − 1 − 1,102, Beckman, United States) were performed. A microplate reader 1705-24B provided by Thermo (Beijing, China) was used.

Hazel mushroom was purchased from Changbai Mountains in Jilin Province, China. The mushroom was dried to a constant weight using a crusher and passed through a 50-mesh sieve. It was then stored at 4 °C.

The following reagents and materials were used in this study: MTT (Amresco, Shanghai, China); apoptosis detection kit (Bioss, Beijing, China); DPPH (Shanghai Ika Biotechnology Co., Ltd., Shanghai, China); *α*-glucosidase, α-amylase, polyphenol oxidase, and lipase (Shanghai Yuanye Biological Co., Ltd., Shanghai, China); and other inorganic reagents (Tianjin Chemical Reagent Company).

In this work A549 human lung cancer cells and MG63 osteoma cells were purchased from Meilun Biotechnology Co., Ltd. (Dalian, China). Newborn bovine serum was obtained from Changchun Sino Biotechnology Co., Ltd. (Changchun, China). DMEM was purchased from Corning Corporation. Water employed in this experiment was of ultra-pure quality.

### 2.2 MAE of total saponins from hazel mushroom

Dry the hazel mushroom in a drying oven at a constant temperature of 45 °C until they reach a constant weight, and grind them with a pulverizer to a diameter of 0.15 millimeters sift and prepare hazel mushroom powder samples, store them in a dry bottle for later use. Approximately 2.0 g of dried hazel mushroom powder was mixed and placed in a microwave vessel (flat-bottomed flask). Subsequently, 40 mL of 70% (v/v) ethanol was concentrated, and these vessels were sealed with ground stoppers. Extraction was carried out using a microwave synthesis/extractor at 200 W for 10 min. The residues were filtered next, and the total saponins in the collected filtrates were measured.

### 2.3 Preparation of sample solutions of hazel mushroom extracts

The dry stive of hazel mushroom (2.0 g) was extracted using 40 mL of 70% (v/v) ethanol at 200 W for 10 min. These extracts were filtered and concentrated by using a rotary evaporator at 55 °C under vacuum. The extracts were dissolved in 100 mL of 80% (v/v) ethanol, placed into a conical flask, and allowed to stand overnight. The resting solution was filtered under reduced pressure to remove impurities, and the solvent was removed by rotary evaporation. About 20 mL of ultra-pure water was used to dissolve the sample, and 20 mL of saturated solution of n-butanol water was added into a 100 mL separating funnel and allowed to stand for 12 h. Thereafter, the n-butanol layer was retained. This solvent was subjected to rotary evaporation. The sample was further dissolved in methanol to a constant volume of 10 mL to determine the saponin content in the solution.

### 2.4 Determination of total saponin content

The total saponin content in hazel mushroom was quantified via a perchloric acid chromogenic approach. SHM was prepared from 2.0 g of material using the established extraction process. After the evaporation of the solvent with a rotary evaporator, 100 mL of ethanol solution (80%, v/v) was added to a conical flask and allowed to stand overnight. The resting solution was subjected to vacuum filtration to eliminate contaminants, and the solvent was removed by rotary evaporation. The sample was dissolved in 20 mL of ultra-pure water and 20 mL of saturated n-butanol–water solution. Subsequently, it was placed in a 100 mL separating funnel and allowed to stand for 12 h. This n-butanol fraction was preserved, and the solvent was eliminated via rotary evaporation. This sample was further dissolved in methanol to a constant volume of 10 mL. A 1 mL sample solution was evaporated to dryness in a water bath. Subsequently, 5 mL of perchloric acid was added, and the mixture was thoroughly homogenized. A hermetically sealed reaction was performed at 65 °C for 15 min. To stop the reaction, we cooled the mixture in an ice-water bath set at 0 °C for an additional 15 min. The sample was then stored at 4 °C for further analysis by using an ultraviolet spectrophotometer. Absorbance readings were conducted at 372 nm via microplate spectroscopy, with perchloric acid as the blank reference. A standard curvilinear equation, with a determination coefficient (R^2^) of 0.9998, was derived from the plot of absorbance (denoted as X) versus concentration (denoted as Y, with units of mg/mL), as outlined in the following Equation 1:


(1) Y=0.4538X−0.1471

The equation indicates that within the saponin concentration range of 0.076–0.38 mg/mL, the absorbance exhibits a well-defined linear relationship with a low margin of error. The saponin content was quantified using Equation 2:


(2) $$Y=\frac{C\times 20\times 20}{m\times 1000}\times 100\%$$

where Y is the saponin extraction rate (%); C is the concentration of this sample (mg/mL); and m is the hazel mushroom quality (g).

#### 2.4.1 Box–Behnken design optimization

Within this optimization work, a three-level, four-factor BBD was effectively utilized. The independent variables for optimizing SHM extraction were solid–liquid ratio (X1), ethanol concentration (X2), extraction time (X3), and extraction power (W). In the experiment, extraction yield (Y) was the response variable. Table 1 shows the coded and actual values of the independent variables. We conducted 29 experiments and used five central-point replicates to assess pure error.

**Table 1:** Factors and levels.

Factor	Low	Center	High
Solid–liquid ratio (g/mL, X1)	−1(1:15)	0(1:20)	1(1:25)
Ethanol concentration (%, X2)	−1(40)	0(50)	1(60)
Extraction time (min, X3)	−1(10)	0(15)	1(20)
Extraction of power (W, X4)	−1(200)	0(300)	1(400)

**−**1, 0, 1 represents coding of level.

After the experiments, a second-order polynomial model was employed to correlate the response variable (extraction yield) with the independent variables. The equation is presented below Equation 3:


(3) $$Y=\beta_{0}+\sum_{i=1}^{k}\beta_{i}x_{i}+\sum_{i=1}^{k}\beta_{\mathrm{ii}}x_{i}^{2}+\sum_{i+1}\sum_{j=i+1}\beta_{\mathrm{ij}}x_{i}x_{j}+\varepsilon$$

Within this framework, Y is the dependent variable; β_0_ is the constant coefficient; β_i_, β_ii_, and β_ij_ are the coefficients for these linear, quadratic, and interaction effects, respectively; *X*_i_ and *X*_j_ represent the independent variables, and epsilon *ε* indicates the error.

### 2.5 UHPLC-Q-TOF-MS analysis

#### 2.5.1 Chromatographic condition

The analysis used an Agilent extended C18 column (2.1 × 50 mm, 1.7 μm) with a positive-charge mode pre-column, on an ACQUITY UPLC/Q-TOF-MS system (ESI). The sample injection volume was 20 μL, flow rate was 0.3 mL/min, column temperature was 35 °C, and detection wavelength was 204 nm. The gradient elution protocol combined mobile phase A (5% aqueous acetic acid) and B (acetonitrile) as follows: 0–1 min, 95% A; 1–10 min, 95–30% A; 10–15 min, 30–20% A; 15–20 min, 20–5% A; 20–28 min, 5–95% A; and 28–30 min, 95% A.

#### 2.5.2 Mass spectrometry conditions

ESI was used for the detection of cations and anions, with a data collection range of m/z 50–1,200. The desolvation temperature was 150 °C, and the gas flow rate was 15 L/min. The protection temperature was 350 °C.

### 2.6 Cytotoxicity and apoptosis

Cells were cultured in DMEM (10% FBS and 1% penicillin–streptomycin) in a humidified incubator at 37 °C and 5% CO_2_.

The viability of control and treated cells was assessed via MTT assay in triplicate, as previously indicated. In brief, the MTT assay was employed to assess the cytotoxicity of SHM. A549 and MG63 cells at a concentration of 1 × 10^5^/mL were seeded into 96-well plates, with each well containing 110 μL of medium. After a 24-h incubation period for cell adherence, the medium was substituted with fresh DMEM. Subsequently, cell cultures were exposed to SHM and incubated for 24 h. Thereafter, DMEM was discarded, and 100 μL of fresh DMEM and 10 μL of MTT solution (5 mg/mL) were added to each well. After 3.5 h of incubation, the supernatants were removed, and 150 μL of DMSO added. The OD was measured at 490 nm using a microplate reader. DMSO administered as the blank control.

A549 and MG63 cells (1.25 × 10^5^/mL) were seeded onto 6-well plates with 2 mL of medium per well. After 24 h, we switched to DMEM. Cells were then treated with SHM (25, 50, 100, and 200 μL) for 24 h to assess apoptosis and cell cycle, using DMSO as a blank control.

An eBioscience Annexin V-FITC Kit was used to detect apoptosis. Cells were harvested, washed twice with cold PBS, and resuspended in binding buffer. After adding Annexin V-FITC and PI, cells were incubated at room temperature for 15 min and analyzed by flow cytometry.

### 2.7 *In vitro* antioxidant profiling of SHM

#### 2.7.1 Determination of hydroxyl radical scavenging capacity

Prepare hazel mushroom saponin solutions with different concentration gradients, take 2 mL of solutions with different concentrations and place them in a ratio. Add 1 mL of 9 mmol/L ferrous sulfate and 1 mL of 9 mmol/L salicylic acid ethanol solution to the color tube in sequence 1 mL of 8.8 mmol/L H_2_O_2_ solution. Start the reaction in a constant temperature incubator at 37 °C and keep it at that temperature for 45 min. Afterwards, distilled water was used as a blank control, and the absorbance of each test solution was measured at a wavelength of 526 nm using a UV spectrophotometer. Brightness, vitamin C as a control, using the same method as above.

#### 2.7.2 Determination of DPPH radical scavenging ability

Prepare a certain concentration of DPPH solution with anhydrous ethanol, dilute to 50 mL in a brown volumetric flask, and store under low temperature and dark conditions for future use. Transfer 2 mL of prepared samples of different concentrations of hazel mushroom saponins to a 10 mL color tube, and transfer 3 mL of DPPH solution to the color tubes containing different concentrations of saponin solutions. Place them in a dark constant temperature box, keep at 50 °C for 60 min, and use 2 mL of distilled water and 3.5 mL of anhydrous ethanol as blank controls. Measure the absorbance at 526 nm and use vitamin C as a control. The method is the same as above.

#### 2.7.3 Determination of total antioxidant capacity

Take 0.4 mL of prepared hazel mushroom saponin solution with different concentration gradients and transfer it to a 10 mL colorimetric tube. Then transfer 4.0 mL of phosphomolybdate test solution (made by mixing 0.6 mol/mL H_2_SO_4_ solution, 28 mmol/mL Na_3_PO_4_ solution, and 4 mmol/mL H_8_MoN_2_O_4_ in a 1:1:1 ratio) to the colorimetric tube. Place the colorimetric tube in a 95 °C constant temperature incubator for 90 min, and cool it down to room temperature after the reaction is complete. Measure the absorbance at 695 nm wavelength using a UV spectrophotometer, use distilled water as a blank control, and use vitamin C solution as a positive control. The absorbance value represents the total antioxidant capacity, and the larger the absorbance value, the stronger the total antioxidant capacity of the sample.

#### 2.7.4 Determination of reducing power

Transfer 2 mL of prepared hazel mushroom saponins with different concentration gradients into a 10 mL colorimetric tube. Then, add 2.5 mL of 0.2 mol/L PBS solution with pH = 6.6 and 2.5 mL of 1% potassium ferrocyanide solution to the colorimetric tube in sequence. React in a 50 °C constant temperature incubator for 20 min, cool to room temperature, and add 2.5 mL of 10% trichloroacetic acid solution to the colorimetric tube. Filter the solution through a filter head to make it clear. Take 2.5 mL of filtered reaction solution and add 0.5 mL of 1% ferric chloride solution and 2.5 mL of distilled water in sequence. React at room temperature for 10 min, measure the absorbance at 700 nm with a UV spectrophotometer, use distilled water as a blank control, and use vitamin C solution as a positive control. The magnitude of the absorbance value represents the magnitude of the reducing force, and the stronger the reducing force, the greater the absorbance value.

## 3 Results and discussion

### 3.1 Effect of different solid–liquid ratios on the saponin yield

**Figure 1:**
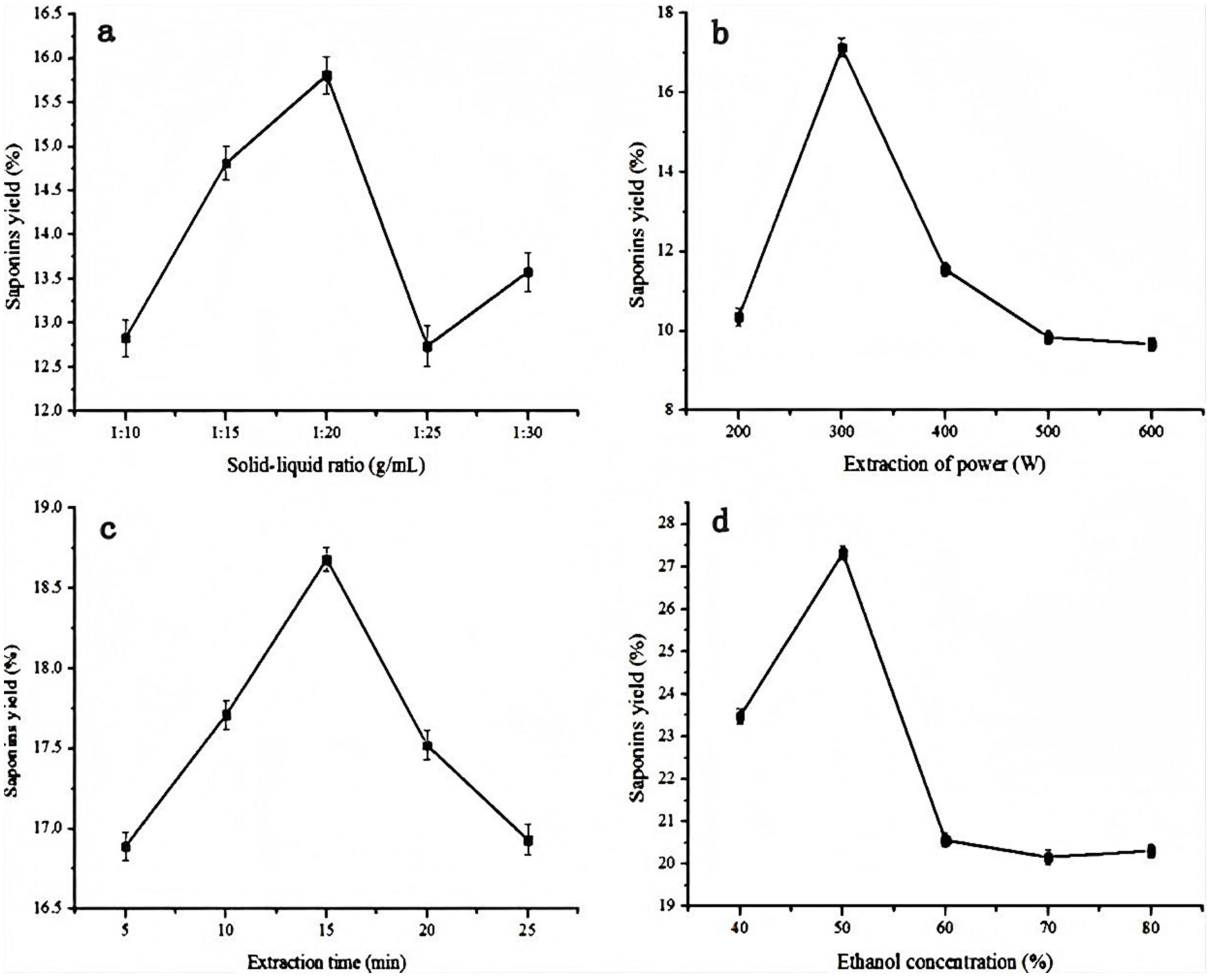
Effects of different **(a)** Solid-liquid ratio, **(b)** Extraction of power, **(c)** Extraction time and **(d)** Ethanol concentration on extraction yield of saponins. Data are shown as the mean ± S.D. from three independent experiments.

The effect of the solid–liquid ratio on saponin yield is depicted in Figure 1a. The solid–liquid ratios were 1:10, 1:15, 1:20, 1:25, and 1:30 g/mL. The other parameters were as follows: extraction power of 300 W, extraction time of 10 min, and ethanol concentration of 70%. As illustrated in Figure 1a, the saponin yield was positively correlated with the solid–liquid ratio, peaking at 15.81% when the ratio reached 1:20 g/mL. A solid–liquid ratio of 1:20 g/mL is adequate for achieving optimal saponin extraction. Consequently, this ratio was designed as the optimal condition for the present test. All experiments were performed in three independent replicates.

### 3.2 Effect of different power extraction methods on the saponin yield

To assess the influence of extraction power on saponin yield, we performed extractions at 200–600 W (increments of 100 W), with a solid–liquid ratio of 1:20 g/mL, extraction time of 10 min, and ethanol concentration of 70%. Initially, the saponin yield rose as the extraction power increased from 200 to 300 W. In Figure 1b, the highest yield (17.13%) was achieved at an extraction power of 300 W. However, when the extraction power increased from 300 to 600 W, the saponin yield declined sharply. Therefore, an extraction power of 300 W was deemed optimal for this study. All experiments were performed in three independent replicates.

### 3.3 Effect of extraction time on the extraction yield of saponins

As illustrated in Figure 1c, we examined how extraction time influenced the saponin yield. In this research, the extraction duration ranged between 5 and 25 min, whereas other parameters remained constant: solid–liquid ratio of 1:20 g/mL, extraction power of 300 W, and ethanol concentration of 70%. These results revealed that a 10 min extraction time yielded the highest saponin content, reaching 18.68%. All experiments were performed in three independent replicates.

### 3.4 Effect of ethanol concentration on the extraction yield of saponins

The saponin yields affected by the ethanol concentration are shown in Figure 1d. Ethanol concentrations were set at 40, 50, 60, 70, and 80%, whereas the other extraction parameters were held constant: solid–liquid ratio of 1:20 g/mL, extraction power of 300 W, and ethanol concentration of 70%. As depicted in Figure 1d, the saponin yield improved with rising ethanol concentration, peaking at approximately 23.47%. Thus, an ethanol concentration of 50% was deemed optimal for this experiment. All experiments were performed in three independent replicates.

### 3.5 Statistical analysis and model fitting

Response surface optimization markedly outperforms conventional single-parameter optimization, notably saving time, space, and raw materials. In this investigation, 29 experimental trials were systematically conducted to optimize four distinct parameters within the Box–Behnken design. Table 2 presents the experimental setups and the resulting saponin yields, as specified by the factorial design. These results indicated that saponin yields ranged from 26.58 to 32.57%. The highest extract value (34.29%) was achieved under the conditions of X_1_ = 25 g/mL, X_2_ = 57.52%, X_3_ = 20.00 min, and X_4_ = 369.75 W. These results were modeled using a second-order polynomial equation. Regression coefficients were derived to elucidate the relationship between the response and experimental variables, as shown in the following second-order polynomial Equation 4:


(4) $$\begin{array}{c} Y=32.25+0.11X_{1}+0.29X_{2}-0.24X_{3}-(7.500E-0.03)X_{4}\\ +0.45X_{1}X_{2}+1.54X_{1}X_{3}+0.20X_{1}X_{4}-0.51X_{2}X_{3}+0.52X_{2}X_{4}\\ +0.92X_{3}X_{4}-1.32X_{1}^{2}-1.80X_{2}^{2}-0.95X_{3}^{2}-2.65X_{4}^{2} \end{array}$$

**Table 2:** Response surface design scheme and experimental saponin yield.

NO.	Solid–liquid ratio X_1_(g/mL)	Ethanol concentration X_2_(%)	Extraction time X_3_(min)	Extraction of power X_4_(W)	Extraction rate (%)
1	1:20	50.00	10.00	200.00	29.44
2	1:20	40.00	10.00	300.00	28.74
3	1:20	60.00	15.00	400.00	28.06
4	1:20	50.00	15.00	300.00	32.26
5	1:15	50.00	20.00	300.00	28.45
6	1:20	60.00	15.00	200.00	27.81
7	1:20	50.00	10.00	400.00	28.21
8	1:25	50.00	15.00	400.00	29.08
9	1:20	40.00	20.00	300.00	29.31
10	1:20	50.00	15.00	300.00	32.57
11	1:20	50.00	15.00	300.00	31.99
12	1:20	40.00	15.00	400.00	26.58
13	1:20	50.00	20.00	400.00	29.48
14	1:25	50.00	20.00	300.00	30.91
15	1:25	50.00	15.00	200.00	28.53
16	1:15	50.00	15.00	400.00	27.99
17	1:15	50.00	10.00	300.00	31.94
18	1:15	40.00	15.00	300.00	29.20
19	1:25	60.00	15.00	300.00	29.76
20	1:20	60.00	20.00	300.00	29.59
21	1:20	60.00	10.00	300.00	31.06
22	1:25	40.00	15.00	300.00	28.88
23	1:20	40.00	15.00	200.00	28.41
24	1:20	50.00	20.00	200.00	27.05
25	1:20	50.00	15.00	300.00	32.26
26	1:15	60.00	15.00	300.00	28.26
27	1:20	50.00	15.00	300.00	32.15
28	1:25	50.00	10.00	300.00	28.25
29	1:15	50.00	15.00	200.00	28.25

The statistical reliability of the regression model was assessed via *F*-test and *p*-value, with the variance analysis for this response surface quadratic model presented in Table 3. The *R*^2^-value (0.9559) confirmed a strong fit for the quadratic model, suggesting its suitability for predicting outcomes within the experimental variable range. *p*-values were utilized to evaluate the statistical significance of each coefficient, with low *p*-values denoting high statistical significance. The model demonstrated significance, as evidenced by a substantial *F*-value (21.70) and a minimal *p* value (below 0.0001).

**Table 3:** Analysis results of variance.

Source	Sum of squares	DF	Mean square	*F*-value	*p*-value
Model	77.30	14	5.52	21.7	<0.0001
X_1_	0.15	1	0.15	0.57	0.4625
X_2_	0.97	1	0.97	3.83	0.0706
X_3_	0.68	1	0.68	2.66	0.1252
X_4_	6.75E-0.04	1	6.75E-0.04	2.653E-0.03	0.9597
X_1_X_2_	0.83	1	0.83	3.25	0.0928
X_1_X_3_	9.46	1	9.46	37.16	<0.0001
X_1_X_4_	0.16	1	0.16	0.64	0.4355
X_2_X_3_	1.04	1	1.04	4.09	0.0627
X_2_X_4_	1.08	1	1.08	4.25	0.0583
X_3_X_4_	3.35	1	3.35	13.16	0.0027
X_1_^2^	11.31	1	11.31	44.45	<0.0001
X_2_^2^	21.03	1	21.03	82.64	<0.0001
X_3_^2^	5.91	1	5.91	23.21	0.0003
X_4_^2^	45.44	1	45.44	178.58	<0.0001
Residual	3.56	14	0.25		
Lack of fit	3.38	10	0.34	7.51	0.0335
Pure error	0.18	4	0.045		
Cor total	80.86	28			
*R* ^2^	0.9559				
Adj*R*^2^	0.9119				
C. V.%	1.71				

This table reveals significant quadratic term coefficients (X_1_^2^, X_2_^2^, X_3_^2^, and X_4_^2^) and an interaction coefficient (X_1_X_3_) (*p* < 0.02); however, the remaining term coefficients (X_1_, X_2_, X_3_, X_4_, X_1_X_2_, X_1_X_4_, X_2_X_3_, X_2_X_4_, and X_3_X_4_) were not statistically significant (*p* > 0.05). Finally, 3D and contour plots were constructed to visually represent and predict the interaction between independent and dependent variables.

**Figure 2:**
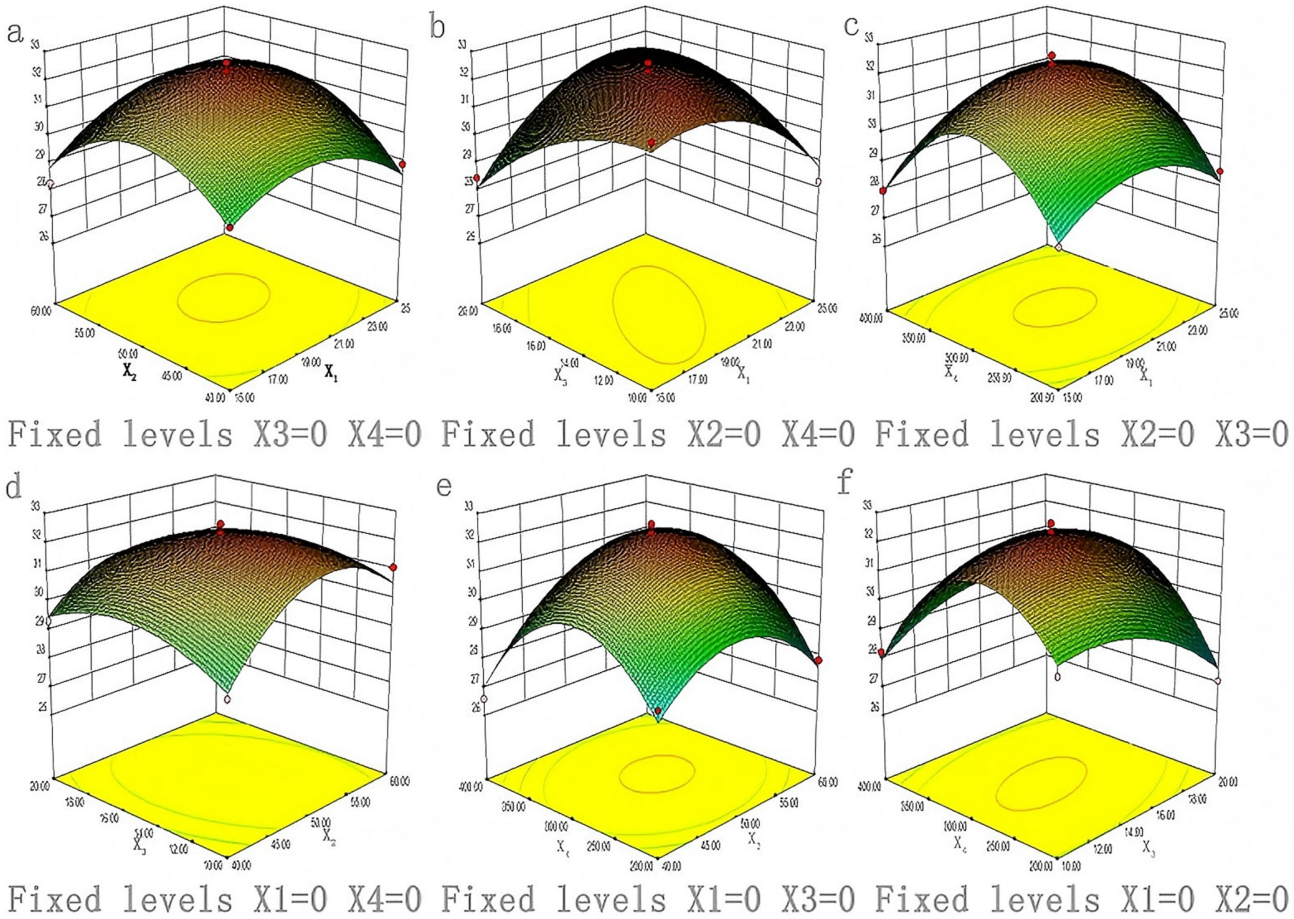
Response surface maps of interaction of different influencing factors.

**Figure 3:**
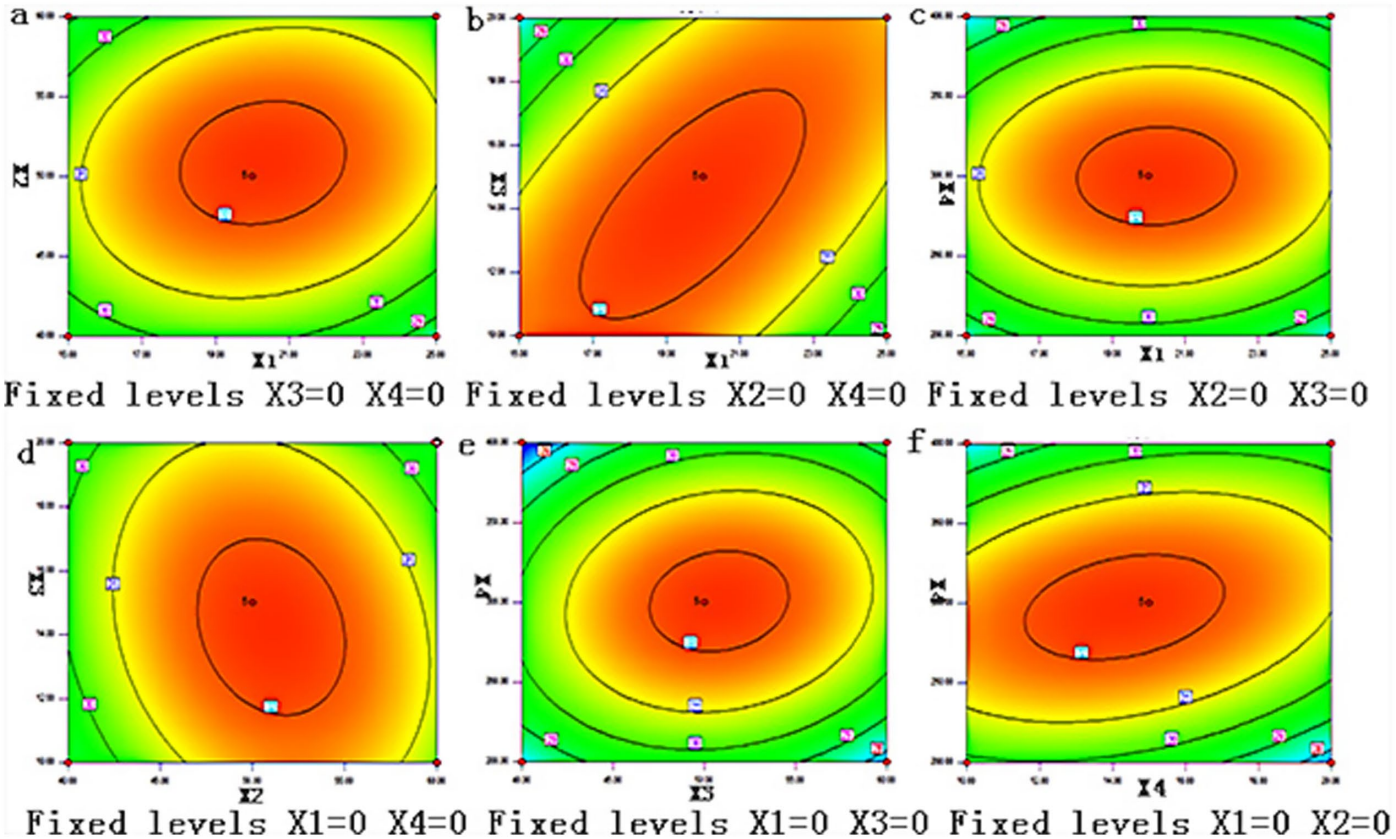
Contour maps of interaction of different influencing factors.

The 3D and contour plots in Figures 2a, 3a show the influence of the saponin yield on the solid–liquid ratio and ethanol concentration (at 300 W power and 10 min), indicating an initial yield rise as the solid–liquid ratio increased from 15 to 21 g/mL. However, beyond 21 g/mL, the yield gradually declined with further increases in the ratio. Additionally, the saponin yield was observed to rise sharply as the ethanol concentration increased from 40 to 50%.

Figures 2b, 3b display the 3D response surface and contour plots, illustrating how variations in solid–liquid ratios and extraction times influenced the saponin yield, while maintaining a constant ethanol concentration (70%) and extraction power (300 W). The results indicated that the saponin extraction yield exhibited a significant increase within the initial 10–14 min of extraction, after which it plateaued, reaching a maximum yield that did not increase further. The yield rose sharply when the solid–liquid ratio grew from 15 to 21 g/mL and then slightly declined when the ratio exceeded 21 g/mL, reaching 25 g/mL.

Figures 2c, 3c show the 3D response surface and contour plots, highlighting the influence of extraction power and solid–liquid ratios on saponin yield, with an ethanol concentration of 70% and extraction time fixed at 10 min. Optimal saponin extraction was reached at 300 W and a solid–liquid ratio of 21 g/mL.

Figures 2d, 3d present the 3D response surface and contour plots for variations in saponin yield with the ethanol concentration and extraction time (solid–liquid ratio of 1:20 g/mL and extraction power of 300 W). These findings indicate that the peak saponin extraction yield was achieved at an extraction time of 18 min and ethanol concentration of 55%.

Figures 2e, 3e show the 3D response surface and contour plots for the saponin extraction yield versus ethanol concentration and extraction power (10 min of extraction and solid–liquid ratio of 1:20 g/mL). These results revealed that the highest saponin extraction yield was achieved at ethanol concentration of 50% and extraction power of 300 W.

Figures 2f, 3f display the 3D response surface and contour plots of the saponin extraction yield versus extraction time and power (solid–liquid ratio of 1:20 g/mL and ethanol concentration of 70%). The results showed that the highest saponin yield was achieved with an extraction time of 18 min and extraction power of 300 W.

The optimal conditions for SHM extraction were an ethanol concentration of 57.52%, solid–liquid ratio of 1:25 g/mL, extraction time of 20.00 min, and power of 369.75 W, the theoretical extraction yield of hazel mushroom saponins under these conditions was 34.29%. In practice, the average yield obtained from three independent experiments was 34.61%, which closely matched the theoretical prediction. These findings suggested that the model was suitable for optimizing the SHM extraction process.

### 3.6 Identification of saponins in SHM

**Figure 4:**
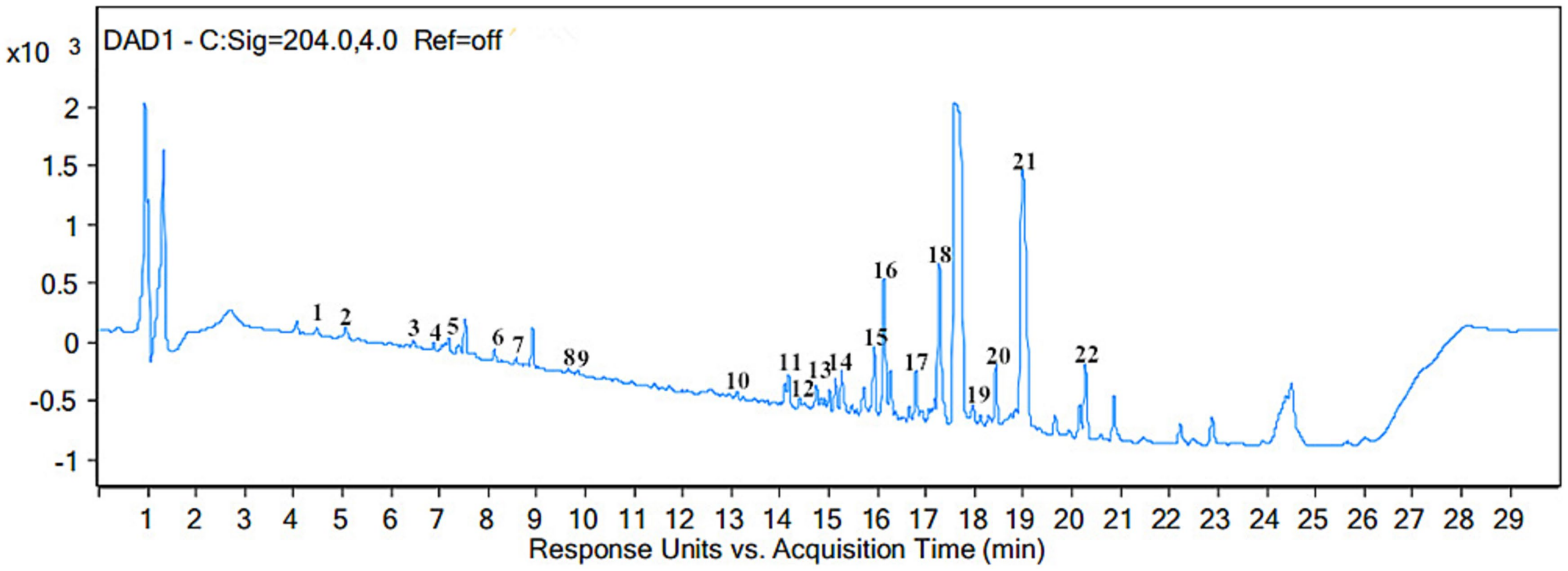
Base peak chromatogram of hazel mushroom saponins obtained by UHPLC-ESI-Q-TOF-MS.

Several analytical methods, such as UV spectrometry, GC–MS, HPLC-DAD, and UHPLC-Q-TOF-MS, are extensively utilized for the identification of saponin compounds. Among these, UHPLC-ESI-QTOF-MS stands out as a highly effective tool for identifying and characterizing known and unknown compounds, utilizing molecular formulas, precise mass measurements, and MS/MS fragmentation patterns. In this study, SHM compounds were characterized via UHPLC-ESI-QTOF-MS/MS under negative ionization. The UHPLC chromatogram for SHM is shown in Figure 4. Table 4 provides a summary of retention times, calculated and detected masses, molecular formulas, mass errors, and MS/MS fragment data. A total of 22 compounds were accurately identified in the MAE extract, comprising 14 hemiterpenes, 3 adenosines, 3 sterols, 1 purine, and 1 terpenoid, by comparing their retention times, exact masses, and fragment ions.

**Table 4:** Analysis of the components in the extract of hazel mushroom saponins.

No	Rt (min)	Detected m/z[M-H]^**−**^	Calculated m/z[M-H]^**−**^	Mass error (ppm)	Formula	MS/MS (MS^E^)m/z	Compounds identification
1	4.696	119.0362	120.0397	−0.0036	C_5_H_4_N_4_	120.0436	Purine
2	5.394	433.138	435.1394	0.0021	C_23_H_27_ClO_6_	434.1479	Melleolide K
3	6.425	395.1849	398.2	−0.0018	C_24_H_28_O_5_	396.1918	Armillaricin
4	6.441	282.0901	283.0842	−0.005	C_10_H_13_N_5_O_5_	283.095	Guanosine hydrate
5	6.691	295.1305	296.1301	0.0005	C_12_H_17_N_5_O_4_	295.1295	N, N-dimethyladenosine
6	7.954	420.2149	422.231	−0.0043	C_23_H_32_O_7_	420.2143	Armillarizin
7	8.104	281.1079	283.1116	0.0026	C_11_H_15_N_5_O_4_	281.1074	6-methyladenosine
8	9.666	431.1734	432.1767	−0.0007	C_23_H_28_O_8_	432.1807	Melledonal A
9	9.766	447.1707	449.1562	−0.0066	C_23_H_28_O_9_	448.1766	Melledonal E
10	12.908	449.1863	451.1768	0.0016	C_24_H_31_ClO_6_	450.1784	Arnamiol
11	14.055	395.3291	398.3347	0.0038	C_28_H_44_O	463.1887	Ergosterol
12	14.405	431.2103		−0.0076	C_24_H_32_O_7_	432.2176	Melleolide B
13	14.621	413.1972	415.2107	−0.0053	C_24_H_30_O_6_	414.2053	4-o-methylmelleolide
14	15.169	402.1974	404.1978	0.0026	C_23_H_30_O_6_	402.1974	Armilly orsellinate
15	16.001	433.1867	435.1904	0.0004	C_23_H_30_O_8_	434.186	Melledonol
16	16.483	411.3237	415.3142	−0.0009	C_28_H_44_O_2_	412.3309	6,9-epoxy-ergosta-7,22-dien-3β-ol
17	16.583	301.2183	303.2269	−0.0056	C_20_H_30_O_2_	302.2256	Sandaracopimaric acid
18	17.929	429.1958	432.1971	−0.0028	C_24_H_30_O_7_	430.2028	Melleolide H
19	17.996	593.4179	595.4323	0.0004	C_38_H_58_O_5_	594.4296	Armillatin
20	18.877	399.1851	401.1888	−0.0012	C_23_H_28_O_6_	400.1912	Melleolide
21	19.043	397.3357	400.3791	0.0014	C_28_H_46_O	398.3486	Ergosta-5,7-dien-3β-ol
22	20.755	383.1904	386.1954	−0.0073	C_23_H_28_O_5_	384.1973	Armillarivin

### 3.7 Cytotoxicity results

Osteosarcoma is the most prevalent primary bone malignancy. It is highly invasive and destructive, primarily affecting children and adolescents. The 5-year survival rate is below 33%, indicating a tumor disease with high mortality. Lung cancer ranks among the most prevalent malignant tumors globally, with increasing incidence each year and a notably low 5-year survival rate, imposing a substantial burden on patients and the healthcare system. Consequently, there is an urgent need to devise effective long-term strategies for cancer prevention and management. In recent years, many plant extracts, such as *Triangularia* and spiral fungus, have been developed as adjunctive drugs for cancer treatment because of their potential anti-tumor activity.

**Figure 5:**
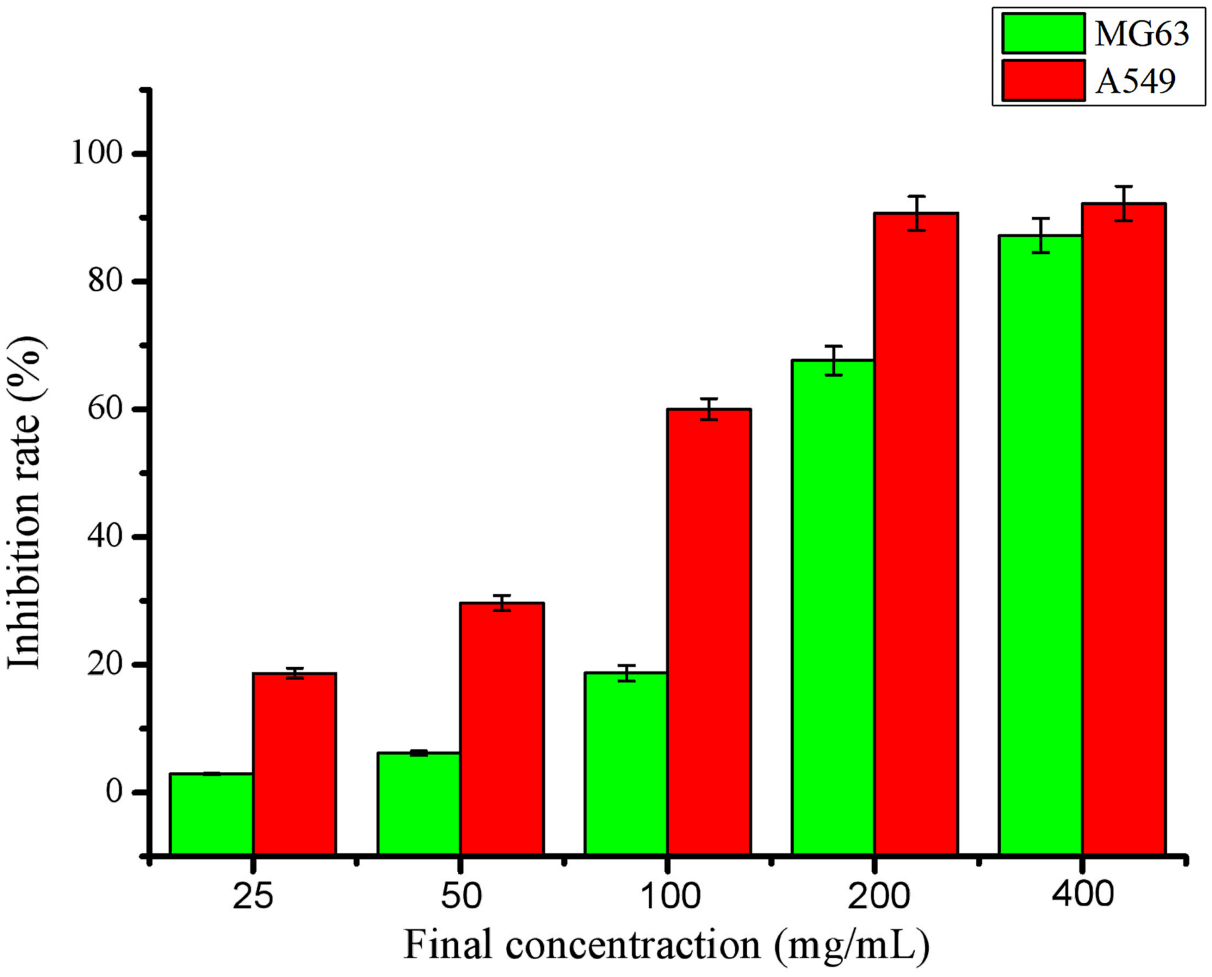
Inhibitory effect of saponins extracted from hazel mushroom on tumor cell MG63 and lung cancer A549.

Therefore, for the first time, our study established *in vitro* assays assessing SHM’s antitumor effects on A549 lung cancer and MG63 osteosarcoma cells. Figure 5 shows that SHM inhibited both tumor cells at 25–400 μg/mL. The inhibitory activity for tumor cells showed a positive correlation with drug concentration. SHM showed better inhibitory activity on A549 cells than on MG63 cells. When SHM reached 400 μg/mL, the inhibition rates of SHM on A549 and MG63 cells reached 93.58 and 87.07%, respectively. In previous reports on the chemical constituents of hazel mushroom, sesquiterpenoids exhibit good anticancer activities. For example, armillaridin inhibits the proliferation of human esophageal cancer cells and enhances radiosensitivity. Armillarikin demonstrates the capacity to suppress the proliferation of human leukemia K562, U937, and HL-60 cells. Combined with the chemical composition analysis of SHM, we hypothesize that sesquiterpenoids in SHM may exhibit antiproliferative effects on A549 and MG63 cells.

The above results indicated that saponins extracted from hazel mushroom effectively suppressed the proliferation of A549 and MG63 cells. This discovery positions hazel mushrooms as promising candidates for the development of targeted functional foods, enhancing their utilization.

### 3.8 Effects of SHM on tumor cell apoptosis

**Figure 6:**
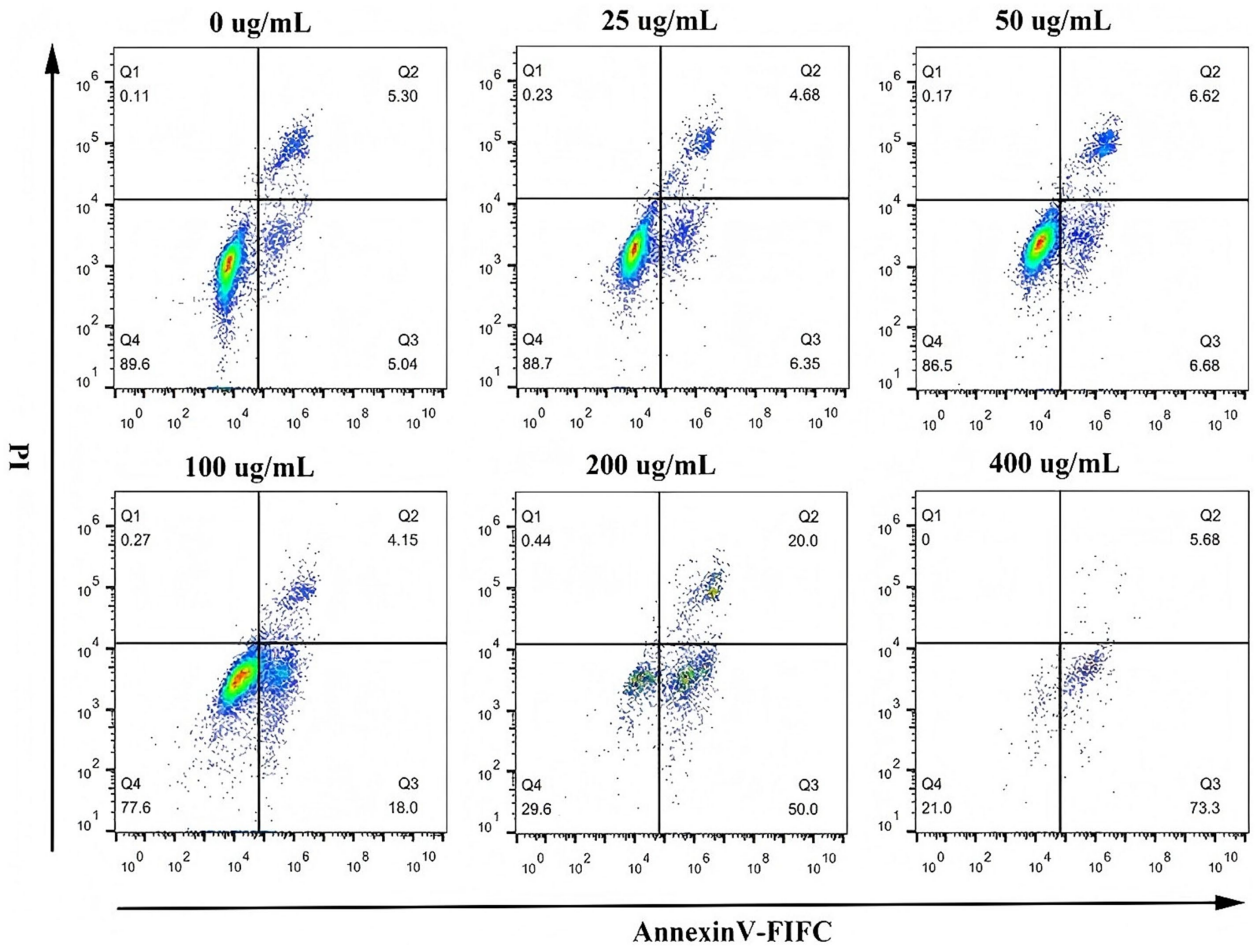
Apoptosis induction in A549 cells by SHM.

**Figure 7:**
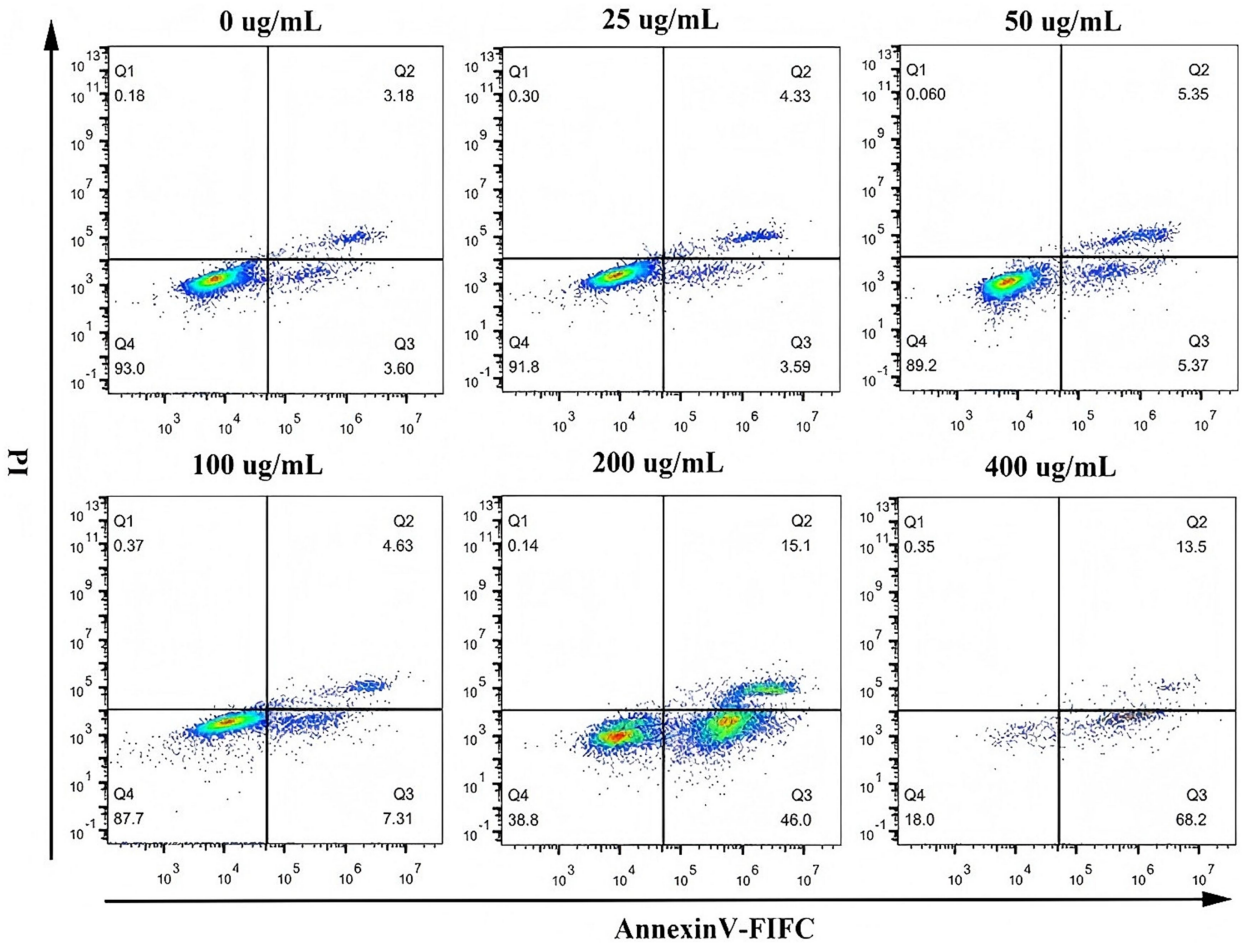
Apoptosis induction in MG63 cells by SHM.

SHM-induced apoptosis in A549 and MG63 cells was assessed by Annexin V-FITC/PI staining and flow cytometry. Figures 6, 7 illustrate a marked decline in the percentage of viable cells with increasing SHM concentrations. Following 24 h of treatment, A549 cells in the blank control group exhibited a basal early apoptosis rate of 6.68%, as determined by Annexin V-FITC/PI staining. The administration of hazel mushroom saponins induced a dose-dependent increase in early apoptosis. A sharp rise to 45.70% was observed at a concentration of 200 μg/mL, which further escalated to 68.60% at 400 μg/mL. After 24 h of treatment, MG63 cells in the blank control group exhibited an early apoptosis rate of 3.60%, as measured by Annexin V-FITC/PI staining. The administration of hazel mushroom saponins induced a dose-dependent increase in early apoptosis. A sharp rise to 46.00% was observed at a concentration of 200 μg/mL, which further escalated to 68.20% at 400 μg/mL. Therefore, SHM could promote the apoptosis of the two tumor cell types, and the effect of apoptosis became more obvious with the increase in the SHM concentration. In addition, we found that apoptosis of both tumor cells occurred at the late stage. According to the literature, armillarixin in hazel mushroom can promote tumor cell apoptosis, and it is classified as a sesquiterpenoid. Notably, 14 half terpenes were identified from SHM. We suspect that SHM may promote apoptosis in tumor cells because of the presence of sesquiterpenoids.

### 3.9 Antioxidant activity results

**Figure 8:**
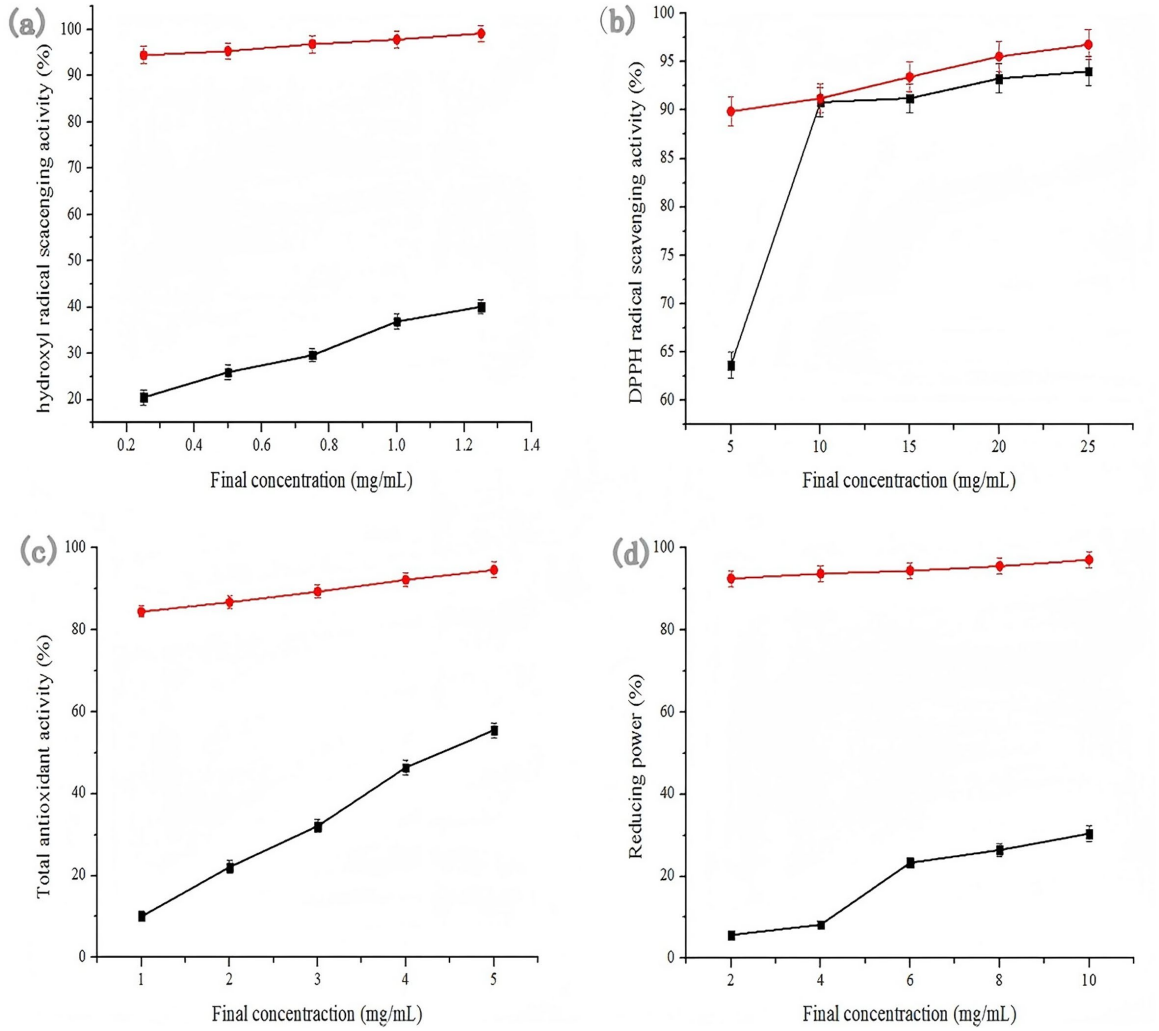
Antioxidant activity of SHM **(a)** hydroxyl radical scavenging activity, **(b)** DPPH radical scavenging activity, **(c)** total antioxidant activity and **(d)** reducing power. Red line: Vc; black line: SHM.

The hydroxyl radical is a strong oxidizer which can stimulate the peroxidation reaction of nucleic acids, protein and lipids. Hence, it is necessary to determine the clearance rate of hydroxyl radical. The results of the assessment of the hydroxyl radical scavenging activity of SHM and Vc at the concentrations ranging from 0.25 to 1.25 mg/mL were observed in Figure 8a. The hydroxyl radical inhibition of the sample and positive control increased at increasing concentrations. The hydroxyl radical anion inhibition of SHM and Vc reached 40.13 and 99.17%, at the concentration of 1.25 mg/mL, respectively. The extracted saponins of hazel mushroom exhibited medium hydroxyl radical scavenging activity.

The DPPH free radical scavenging assay was widely used for evaluating the activity of natural antioxidant, because DPPH radical scavenging assay of SHM was measured at the absorbance of 526 nm and inhibition rates were performed in Figure 8b. The saponins of SHM and positive antioxidant (Vc) displayed clear dose-dependent at the concentrations in the range of 5–25 mg/mL. As the concentration increased from 5 to 10 mg/mL, the DPPH scavenging activity of SHM was rapidly enhanced. However, the DPPH free radical inhibition rate gradually flattened out when the concentration at the range from 10 to 25 mg/mL. The highest DPPH radical cation inhibitions of SHM and Vc were inspected at 25 mg/mL, which were 94.01 and 96.78%, respectively. It was obvious that Vc had better antioxidant than that of the samples at all tested concentrations. The results indicated that the saponins extracted from hazel mushroom had significant scavenging activity of DPPH radicals.

The total antioxidant activity was determined by the modified Prieto method. The determination method of phospho-molybdenum complex is based on the principle that Mo (VI) is reduced to green Mo (V) by antioxidants, the total antioxidant activity of SHM was measured at the absorbance of 695 nm and inhibition rates were performed in Figure 8c. The SHM and positive antioxidant (Vc) displayed clear dose-dependent at the concentrations in the range of 1–5 mg/mL. As the concentration increased from 1 to 5 mg/mL, the total antioxidant activity of SHM was rapidly enhanced, but positive control Vc showed a better total antioxidant capacity in the range of 1–5 mg/mL. The total antioxidant activity of SHM and Vc reached 55.5 and 94.6%, at the concentration of 5 mg/mL, respectively.

Reducing power is closely associated with antioxidant activity and is typically analyzed by absorbance at 700 nm. As depicted in Figure 8d, the reducing power of the sample and positive control increased at increasing concentrations. The reducing power of SHM and Vc reached 30.4 and 97.06%, at the concentration of 10 mg/mL, respectively. Besides, the reducing power of these saponins were still lower than that of Vc in solutions of concentration 2–10 mg/mL. SHM showed lower reducing power than VC, which was possible due to fewer phenolic components. These experiments demonstrated that the SHM can act as antioxidant due to their abilities to act as electron and hydrogen donors to terminate radical chain reactions. Hazel mushroom saponins demonstrate antioxidant activity, which may impart anti-fatigue and neuroprotective effects through the protection of nerve cells. Additionally, their antioxidant capacity likely contributes to anti-inflammatory effects by mitigating systemic oxidative stress.

## 4 Conclusion

In this work, we investigated the extraction and purification techniques, chemical composition, and *in vitro* anti-tumor activity and antioxidant activity of saponins derived from hazel mushroom. Optimal MAE parameters were determined via single-factor and response surface tests: ethanol concentration of 57.52%, 20 min of ultrasonic extraction, solvent-to-material ratio of 1:25 g/mL, and power of 369.75 W, resulting in a 34.61% extraction rate. MAE was more efficient than conventional methods, supported by yield improvement data. Furthermore, the primary constituents of SHM were preliminarily isolated and characterized by UPLC/Q-TOF-MS, providing crucial insights for the pharmacodynamic study of hazel mushroom. SHM demonstrated significant pharmacological efficacy by inhibiting tumor cell proliferation and promoting apoptosis. SHM displayed a certain degree of antioxidant activity. It is important to acknowledge the limitations of the present research. Lacking of *in vivo* confirmation or chemical stability analysis of SHM saponins might be one of the limitations. MAE-based extraction may enable scalable production of SHM-derived bioactives for functional foods. Our study facilitated the exploration of food with targeted functionality.

## Data Availability

The original contributions presented in the study are included in the article/supplementary material, further inquiries can be directed to the corresponding authors.
